# Pig vaccination strategies based on enterotoxigenic *Escherichia coli* toxins

**DOI:** 10.1007/s42770-021-00567-3

**Published:** 2021-07-10

**Authors:** J. Daniel Dubreuil

**Affiliations:** Department of Pathology and Microbiology, Faculté de Médecine Vétérinaire, Université de Montréal, 3200 rue Sicotte, Saint-Hyacinthe, Québec J2S-2M2 Canada

**Keywords:** ETEC, Vaccination strategies, Enterotoxins, Pig diarrhea

## Abstract

Enterotoxigenic *Escherichia coli* (ETEC) are responsible for diarrhea in humans as well as in farm animals. ETEC infections in newborn, suckling, and especially in post-weaning piglets are associated with reduced growth rate, morbidity, and mortality. ETEC express virulence factors as adhesin and enterotoxins that play a central role in the pathogenic process. Adhesins associated with pigs are of diverse type being either fimbrial or non-fimbrial. Enterotoxins belong to two groups: heat-labile (LT) and heat-stable (ST). Heterogeneity of ETEC strains encompass expression of various fimbriae (F4, F5, F6, F18, and F41) and enterotoxins (LT, STa, STb, and EAST1). In the late years, attempts to immunize animals against neonatal and post-weaning diarrhea were focused on the development of anti-adhesin strategies as this is the initial step of ETEC pathogenesis. Although those vaccines demonstrated some protection against ETEC infections, as enterotoxins are pivotal to the virulence of ETEC, a new generation of vaccinal molecules, which include adhesin and one or more enterotoxins, were recently tested. Some of these newly developed chimeric fusion proteins are intended to control as well human diarrhea as enterotoxins are more or less common with the ones found in pigs. As these could not be tested in the natural host (human), either a mouse or pig model was substituted to evaluate the protection efficacy. For the advancement of pig vaccine, mice were sometimes used for preliminary testing. This review summarizes advances in the anti-enterotoxin immunization strategies considered in the last 10 years.

## Introduction

Diverse types of *Escherichia coli* have been described and these show distinct patterns of illness as well as different virulence factors. Among *E. coli* pathogens, we have the following categories: enteropathogenic *E. coli* (EPEC), enterohaemorrhagic *E. coli* (EHEC), enteroaggregative *E. coli* (EAEC), enteroinvasive *E. coli* (EIEC), diffusely adherent *E. coli* (DAEC), and enterotoxigenic *E. coli* (ETEC) [1].

Enterotoxigenic *Escherichia coli* (ETEC) are a common cause of diarrhea in farm animals [2]. Within farm animals, ETEC infections are mainly associated with neonatal cattle and piglets. ETEC infections in pigs during the post-weaning period hamper growth and is associated with an increased mortality rate leading to important economic losses worldwide [3]. The key virulence factors of ETEC are adhesins also called colonization factors and enterotoxins. The number of lineages containing porcine ETEC is limited suggesting that a specific chromosomal background is required to harbor ETEC plasmids related to virulence [3]. Thus, specific combinations of ETEC virulence factors are required in the appropriate phylogenetic background to enhanced virulence.

Adhesins play a critical role in the pathogenesis of ETEC strains. The infection spread through the fecal–oral route, and following ETEC ingestion, colonization of the intestine is observed. Adhesion, a prerequisite to colonization, results from the interaction of fimbrial or non-fimbrial adhesins occurring through and only if specific receptors are present on the apical side of cells of the small intestinal epithelium [3]. In pig-specific ETEC strains, five fimbrial (F4 (K88), F5 (K99), F6 (987P), F41, and F18) and one afimbrial adhesin (adhesion involved in diffuse adherence, AIDA) have been identified [4].

ETEC can produce and deliver heat-labile (LT) and/or heat-stable (STs) enterotoxins. In pigs, three STs are produce: STa, STb, and EAST1 [5]. Upon attachment of these toxic molecules to specific receptors for each toxin present on the intestinal epithelium, an alteration of the electrolyte homeostasis results contributing a fluid loss and eventually a secretory diarrhea.

Vaccination considered the most efficient and practical way to reduce the impact of ETEC diarrhea could impede at diverse level the pathogenesis process. Thus, the first step in ETEC pathogenesis is the adhesion process, and for this reason, the first generation of vaccine has been mainly oriented to obtain protection through immunological stimulation toward these antigens. Blocking this essential process should avoid colonization of the intestine and thus in situ elaboration and concentration of enterotoxins and the complications thereof. In the last decade, studies have focused on construction of multiple adhesin fusion proteins for human or animal immunization [6, 7]. For human vaccine testing, a pig model is often used as piglets are naturally susceptible to ETEC infection and develop clinical diarrhea similar to human patients experiencing ETEC infection.

Epidemiological studies have revealed that many of the porcine ETEC strains harbor multiple enterotoxins but actually lack any of the known fimbrial or non-fimbrial adhesins [8]. Thus, the existing commercial vaccines do not provide broad protection against ETEC strains encountered in the field. Although adhesin-mediated colonization is critical in ETEC pathogenesis, enterotoxins as direct virulence factors causing diseases can also be involve in the colonization process itself [9]. These are reasons why a vaccine aiming directly at ETEC enterotoxins is desirable.

As the distribution of enterotoxins vary from region to region and over time in a region [8], efforts were directed at the development of a polyvalent vaccine designed to ideally stimulate an immunological response against all enterotoxins as well as currently observed adhesins [10]. This implies that a multivalent ETEC vaccine is required. There is evidence that a strong mucosal antibody response with secretory IgA immunoglobulin (sIgA) production is needed for prevention of ETEC diarrhea [11].

A vaccinal approach based on adhesins and specifically LT was investigated. Previous studies had shown that LT could clearly bring a protective immune response whereas, due to their small sizes, STa and STb are poorly immunogenic [12, 13]. One problem with the enterotoxins is to obtain toxoids that retain the attribute to stimulate an immune response while being non-toxic. Detoxified LT molecules and the non-toxic B subunit of LT (LTB) have been effectively used as immunogens to induce protective antibodies against heat-labile toxin [7]. For STa and STb, various ways to increase the immunogenicity of the vaccine components were studied and their detoxification has hampered the development of vaccinal molecules. The vaccination objective to obtain neutralizing antibodies able to protect young pigs against ETEC infection have been tested in a mouse model or directly in the normal host, the pig.

In this review, we will focus on the vaccination strategies relying on enterotoxins that have been investigated to protect pigs. As enterotoxins are common to human ETEC strains, some studies were also pursued at the same time to develop a vaccine for human vaccination purpose.

## Diarrhea in pigs

### Neonatal diarrhea

The gut of the newborn pig is sterile but is rapidly colonized by bacteria, including *E. coli*. Neonatal diarrhea occurs in piglets of 0–4 days of age. Antibodies in the colostrum, and later in milk, protect against the damaging effects of ETEC [2]. The sources of infection are piglets, the environment, and feces of the sows. The usual causes are ETEC that possess F4, F5, F6, or F7 adhesins [3, 4, 14].

### Post-weaning diarrhea

Piglets are weaned at around 3–4 weeks of age. Actual vaccines provide incomplete protection against post-weaning diarrhea (PWD) ETEC infections in piglets [15]. One of the reason is probably due to the loss of protective antibodies in piglets receiving colostrum. The absence or low level of antibodies in the intestinal lumen post-weaning leads to a lack of protection against colonization by ETEC. Maternal protection does not extend beyond the suckling period as passively acquired antibodies are rapidly cleared. Therefore, post-weaning piglets are naïve to ETEC infection. In PWD, the fimbriae more often encountered are F4 and F18.

For approaches on immunization strategies based on anti-adhesin approach and problems related to ETEC-mediated PWD in piglets, you can refer to recently published reviews [16–18].

## Adhesins

ETEC adhere to intestinal cells through proteinaceous appendages called fimbriae that they express [4]. Afimbrial adhesion also exist and play an identical role in the pathogenic process. Those molecules bind to specific receptors present on the small intestine epithelium [19]. Once bound, the bacteria colonize the intestinal tissue.

Diarrhea in pigs rely on five fimbrial appendages: F4 (K88), F5 (K99), F6 (987P), F7 (F41), and F18 [20]. The most frequent fimbriae associated with diarrhea and mortality in newborn, suckling, and newly weaned piglets is F4. F18 is commonly associated with PWD whereas F5, F6, and F7 are associated with neonatal diarrhea [3].

F4 and F18 fimbriae present diverse antigenic variants. F4 can be of three types: F4ab, F4ac, and F4ad with F4ac variant being the most prevalent. Two variants of F18 exist, namely F18ab and F18ac, related largely to PWD. Afimbrial adhesion involving AIDA was described [21].

Very early, in vitro and in vivo studies confirmed that fimbriae are highly immunogenic and that they could induce protecting antibodies inhibiting adhesion to enterocytes and colonization of the intestine [3]. The binding of fimbriae to their respective intestinal receptors is critical for the activation of mucosal immunity after oral immunization [22, 23]

Colostral antibodies induced following maternal immunization protect neonatal piglets. Based on this information, development of anti-fimbrial vaccines based on various fimbrial proteins was shown to be effective in protecting animals from neonatal diarrhea. Nevertheless, frustration persisted as the current vaccine preparations based on colostral immunity were not efficient in preventing PWD. Although antagonistic types occur, cross-reactivity of the major and minor structural subunits of F4 was observed. This results in protection regardless of the origin of the F4 present in the vaccinal preparation. However, there was no cross-reactivity observed between F4, F5, F6, and F41.

## Enterotoxins

Enterotoxins produced by ETEC are responsible for inducing diarrhea in the animals. These affect the water-electrolyte balance in the intestine resulting in a secretory diarrhea. They include heat-labile toxin (LT) and heat-stable toxins (STs) with STa, STb, and the enteroaggregative heat-stable toxin 1 (EAST1). These enterotoxins, beside EAST1 that was originally described in an enteroaggregative *E. coli* isolate, are specific to ETEC [24]. Isolates responsible for diarrhea in pigs produce various combinations of these toxins [5].

### LT

LT (85 kDa) is a AB_5_-type toxin consisting of an enzymatic toxic subunit (LTA1), a A2 subunit (LTA2), and five polypeptidic B chains (LTB) that are involved in receptor recognition and binding to ganglioside GM1, a molecule present on the intestinal epithelium [5]. This toxin is capable of inducing systemic and mucosal antibodies. LTB is a potent immunogen and has been regarded as the best adjuvant in eliciting host mucosal immunity who play a key role in protection against enteric infection [25]. LT is also directly involved in the colonization process [9]. LT has been used to increase immunogenicity of STa and STb in vaccines [26]. A laboratory-developed mutant (LT_R192G_) was largely used as a safe antigen to induce anti-LT immunity to protect pigs as it shows no toxicity [27–29]. Other LT non-toxic mutants were also developed. Many vaccines were designed targeting either LT-STa or LT-STb. However, these constructions by themselves could not prevent completely diarrhea caused by ETEC where LT, STa, and STb enterotoxins are altogether produced.

### STa

STa (≈ 2 kDa, 18 aa (STaP) or 19aa (STaH)) is a toxin of peptidic nature comprising 3 disulfide bonds and presents a poor immunogenicity [30]. STa binds to guanylate cyclase C (GC-C) and an elevation of cGMP levels ultimately results leading to secretory diarrhea [31, 32]. ETEC-infected neonatal pigs with strains expressing STaH or STaP (or LT) develop the same clinical signs [33]. STa can cause diarrhea unless it is truncated or modified. Full-length of STa mutants including STaP_N11K_, STaP_P12F_, STaP_A13Q_, and STaH_P14Q_ were tested and shown to be not toxic [28, 34].

### STb

STb (5.2 kDa, 48aa) is poorly immunogenic but this characteristic can be enhanced by fusion to a highly immunogenic carrier molecule [13]. This toxin comprises two disulfide bonds. Its receptor is a glycoshingolipid called sulfatide [35]. The gene coding for STb is highly prevalent in ETEC strains isolated from pigs with PWD [36–38]. In fact, STb-positive ETEC strains are more prevalent than STa in Canada [39], Poland [40], and Spain [41].

### EAST1

EAST1 (4.1 kDa, 38aa) shows homology to STa and it shares the same receptor [42]. It has 50% homology with the enterotoxic domain of STa and is found in human strains but also in *E. coli* strains associated with pig diarrhea [43]. It could also be responsible for diarrheal disease in man. The mechanism of action of EAST1 was proposed to be identical to that of STa eliciting a cGMP increased [42].

For more details on ETEC toxins, you can refer to the following reviews: [3, 42].

## Designing a vaccine

In order to produce a safe vaccine, some measures have to be taken, especially with potent toxic molecules. Among these requirements, molecules that we need to modify should retain their immunogenicity [30, 44, 45]. In the case the antigens are poorly or not at all immunogenic, at least two approaches can be pursued. Coupling of a poor immunogen to a carrier molecule can result in a stronger immunogenic response. This can also be attained through the use of recombinant molecule. In addition, compared to coupling reactions, recombinant techniques have the advantage of being a simple and inexpensive way to produce toxoids. These chimeric proteins can either be delivered by live bacterial vectors [46] or produced for parental immunization of animals. The term parenteral injection encompasses various administration route: intravenous (IV), intramuscular (IM), subcutaneous (SC), intradermal (ID), and intraperitoneal (IP). Detoxification of enterotoxins is also pivotal to the production of a safe vaccine and various ways can be taken to reach this goal [18, 47].

The induction of an immunological response against STa and STb has merit as these two toxins are frequently associated with ETEC strains. STs are not immunogenic and hence coupling by chemical conjugation to an appropriate carrier is required. STa can also be chemically synthetize and then coupled to a carrier molecule [48, 49]. Detoxification of STs can result from genetic fusion or chemical conjugation often coupled with mutagenesis. It is important to understand that lack of STb in current immunization program may result in the blooming of STb-dependent colibacillosis in the future [50].

In peculiar, the main challenges for making a vaccine incorporating ST enterotoxins were to engineer molecules that retain their immunogenicity while reducing or abolishing toxicity. Such mutants are referred as toxoids. For example, due to its small size, separating toxicity from the antigenicity of STa was hard to achieve. Considering STa, another obstacle for STa vaccine development was to construct an immunogen that do not elicit antibodies that could react with guanylin and uroguanylin, due to structural similarities, which are endogenous peptides regulating the activity of guanylate cyclase-C receptor [48]. For example, Diaz et al. (2019) immunized five mice each with both native human STaH and porcine STaP chemically coupled to bovine serum albumin [45]. The sera from the animals neutralized the toxic activities of both STaH and STaP. However, all mouse sera, except two, demonstrated cross-reaction with the endogenous peptides. This showed that STa nucleotide sequence must be precisely mutated in order to reduce the cross-reactivity observed. These researchers also produced four anti-STaH and six anti-STaP monoclonal antibodies. Of all the monoclonal antibodies tested, only one displayed cross-reactivity to the endogenous peptides, suggesting that mutations of a limited number of STa residues could be sufficient to obtain a safe STa vaccine.

Labrie et al. (2001) performed a structure–function study where multiple STb mutants were obtained. Single and double point mutations permitted to determine a way to detoxify, at least partly, this enterotoxin [51]. Many of these mutants were recognized by a rabbit antiserum raised against native STb; nevertheless, none of these was tested for their immunogenicity in an animal model.

On the other hand, LT is a good immunogen, and as such, LTB, immunogenic in many animals, including mice, rats, rabbits, and pigs, is an attractive carrier molecule as, at the same time, it results in immunization against LT. This toxin is also a recognized mucosal adjuvant and is responsible for adhesion of ETEC to the intestinal epithelium [9]. A non-toxic mutant has been identified (LT_R192G_) and is commonly used in vaccinal trials. Genetic fusion of LT_R192G_ with ST antigens was shown to enhance anti-ST immunogenicity and elicited protective anti-LT and anti-ST immunogenicity [28, 34].

## Oral vaccines

Piglet diarrhea is responsible for huge economic loss to the pig industry, and currently, vaccination is the most effective way of controlling ETEC diarrhea [47]. Subunit vaccines delivered by injection suffer from the fact that large dose and repeated administration are required. Stressing of animals also constitute a drawback [52]. To overcome these issues, development of oral vaccines to deliver heterologous antigens was proposed. Oral vaccines are inexpensive to manufacture [53], easy to administer, safe, adequate for large-scale usage, and stable without refrigeration when lyophilized [54].

The gastrointestinal (GI) tract is the animal largest immunological organ. It produces daily more than 60% of the animal antibodies [55]. There is evidence that a strong mucosal antibody response with sIgA production is needed to protect against ETEC diarrhea [11]. Moreover, mucosal surface of the GI tract is the gateway of ETEC. Activation of secreted intestinal anti-ETEC response is impossible to achieve by parenteral administration [56]. Also, secreted IgA represent the first line of defense against invasion of deeper tissue by some pathogens as well as it is able to neutralize the secreted enterotoxins. Therefore, oral delivery is a natural approach through presentation of the antigens to the gut-associated lymphoid tissue.

Oral administration of immunogenic molecules to sows before farrowing result in maternal immunity that could be passively acquired by piglets through ingestion of the colostrum. This protects the animals for about 1 week under normal farming conditions [2]. Therefore, an effective and universal protective vaccine against PWD is lacking [15]. In North America, the majority of piglet-causing diarrhea ETEC express F4ac or F18 fimbriae [57]; for that reason, fimbriae-based vaccines were foreseen in an early stage of vaccine development. Oral immunization of weaned piglets with F4 and F18 was tested and shown to be better at obtaining a mucosal response than IM injection. However, in other countries, as for example China, these fimbriae were not frequently associated with ETEC. In the Netherlands [58], Japan [59], and Sweden [60], fimbriae were rarely associated with ETEC in piglets establishing that vaccine based solely on adhesins could be less effective, and thus, vaccines comprising enterotoxins antigens should be investigated.

To obtain a live attenuated vaccine, some studies reported expression of a multivalent adhesion-toxoid fusion antigen in an avirulent *E. coli* isolate. Recent work showed that simultaneous oral immunization of mice with live recombinant attenuated *E. coli* (expressing LT_R192G_-STa_A13Q_ and LT_R192G_-STb fusion immunogen) could represent an efficient carrier for delivery of a vaccine by the oral route [61]. An advantage is that such a recombinant *E. coli* vector can deliver antigens to the immune system for a prolonged period. This specific preparation demonstrated, for the first time, that it could deliver ETEC enterotoxins simultaneously, and that even at high dosage (10^9^ CFU), no toxicity reaction was induced. In addition, this construction was shown to be stable for over 100 generations.

In a study by Ruan and Zhang (2013), an adhesion-toxoid fusion protein was expressed as an LT-like, that is, a 1A:5B construction, consisting of LTB subunits forming independently a pentamer and where a LTA-like element could independently associate to the formed pentamer [15]. This chimeric protein was secreted by an *E. coli* strain and could bind to GM1, its natural receptor, through the LTB pentamer. The construction consisted also of the major subunit of F4 (FaeG) and a minor component of F18 (FedF) fimbriae. This chimeric protein, consisting of 1FaeG-FedF-LT_R192G_A2:5LTB inserted into an avirulent *E. coli* strain, was evaluated for its vaccine potential when given orally to piglets. The animals developed a systemic and a mucosal response and the resulting colonization of the pig small intestine by this *E. coli* strain induced antitoxin and anti-adhesin mucosal immunity protecting piglets against PWD. The antibodies were able to neutralize CT (a structural and mechanistically based analog of LT) and inhibited F4 and F18 adherence in vivo. No adverse effect was noted following administration of this recombinant strain. Challenged piglets with a virulent ETEC strain (F4ac/LT/STb) showed less colonization of the small intestine and the animals did not develop diarrhea compared to controls who developed diarrhea and died establishing this fusion protein as a good ETEC vaccine candidate.

As STb is a virulence determinant in porcine PWD, it is clearly an asset to include this antigen in a vaccine. An unpublished study had shown that a live *E. coli* strain expressing a F4ac fimbriae and a toxin fusion, LT_R192G_-STb, brought protective immunity against infection with a F4ac/ LT/ STb-positive ETEC strain [62]. A late study where piglets were orally immunized with this live attenuated strain compared to an IM injection showed significantly greater titers of anti-F4ac sIgA in fecal and intestinal washes and anti-LT IgA in intestinal washes. This constituted the first report of a multivalent oral vaccine focusing on the porcine ETEC enterotoxins concurrently that included STb. The adopted strategy permitted a successful colonization by the vaccinal strain, resulting in an effective immunological response.

A study directed at evaluating the immune effect of two live attenuated F41-positive *E. coli* strains expressing LT_R192G_-STa_A13Q_ and LT_R192G_-STb fusion immunogen orally administrated was performed by Liu et al. [61]. Local mucosal and systematic immune responses against LT, STa, STb, and F41 were induced in BALB/c mice immunize with both vaccinal strains. The stimulation index (SI) values evaluating the cellular immune response were significantly higher than the control mice (p < 0.05). A marked shift toward type-2 helper T lymphocyte (Th2) immunity was reported. In that study, the chimeric fusion proteins LT_R192G_-STa_A13Q_ and LT_R192G_-STb retained their native LT promoter, nucleotides coding two ribosome binding sites of LTA and LTB subunits. Thus, the antigens were expressed without need of induction and could directly stimulate the mucosal immune system of the GI. This stimulation lasted a long time after oral administration representing a clear advantage. Oral administration was also capable of inducing a systemic immune response with IgG production. In vitro and in vivo neutralization assays confirmed the immune efficacy of the induced antibodies in inhibiting LT, STa, and STb enterotoxins. In addition, inhibition of STa and STb enterotoxins were observed in situ in suckling mice. Mice fed mother colostrum showed protection compared to control group clearly indicating that this vaccinal strategy elicited neutralizing antibodies against LT, STa, and STb enterotoxins.

You et al. (2011) evaluated a trivalent enterotoxin fusion protein (STa-LTB-STb) [63]. Mice immunized with this construction elicited significant antibody response against the three enterotoxins. In an ETEC challenge, the immunized group showed a survival rate of 70%. Mice were also immunized with a killed preparation of an *E. coli* F4ac strain and the solubilized fusion protein. In a challenge, only 20% of those animals survived. A lack of antibodies against LT, STa, and STb may have contributed to the relatively low survival rates of these orally immunized group or it may be the result from the possible degradation of surface antigen of the killed *E. coli* F4ac bacteria due to the thawing-freezing cycles and/or the low expression level of F4ac fimbriae. This experiment indicated that oral immunization with a killed bacterial ETEC strain can also suffer from major drawbacks.

Feng and Guan (2019) designed a recombinant *E. coli* strain expressing LTA-STa_A13Q_-STb-LTA2-LTB-STa_A13Q_-STb fusion antigen [64]. Since the LT promoter was preserved, the expression of the fusion protein did not require an inducer. Oral immunization with this molecule, containing all ETEC enterotoxins, elicited a potent systemic and mucosal response in a mouse model. IgG ELISA titers were statistically different in spleen, milk, mesenteric lymph nodes, and intestinal mucus of immunized mice. These had also a higher level of IL-4 than IFN-ϒ, suggesting a Th2-oriented response.

## Parenteral immunization

With time, it became clear that effective ETEC vaccines needed to induce both anti-adhesin immunity to block adherence and anti-toxin immunity to neutralize enterotoxicity [44, 65]. A major question still remained: should we favor oral immunization over parenteral immunization?

Taking into account that STa becomes immunogenic only after coupling with an immunogenic carrier and that we need to detoxify this toxin, Zhang et al. (2010) genetically mutated porcine LT gene (pLT_R192G_ toxoid) and the porcine STa gene to obtain three full-length toxoids (STa_N11K_, STa_P12F_, and STa_A13Q_) [34]. The full-length pLT_R192G_ was used as an adjuvant to carry the STa toxoid fusion antigens (LT_R192G_:STa). The data for STa toxoids STa_P12F_ and STa_A13Q_ with only one amino acid replacement showed that both toxoids were recognized by anti-STa antibodies, indicating that these two toxoids had no major structure alterations. These were not able to stimulate fluid secretion in porcine gut loops, diarrhea in gnotobiotic piglets, or an increase in cGMP levels in T84 cells. These two toxoids likely retained very low toxicity (active at more than 1000 × -fold of each peptide). Following intramuscular immunization, rabbits developed high titers of anti-LT and anti-STa antibodies. Rabbit antiserum and fecal antibodies were able to neutralize pure CT (an analog of LT) and STa toxoid. Suckling piglets born from immunized sows were protected from a challenge with a STa-positive ETEC strain. Preliminary data from an animal challenge study showed that three out of four piglets were protected against infection with a STa-producing ETEC strain.

Seo et al. (2019) immunized mice with STa toxoid fusion and chemical conjugates [66]. Mice subcutaneously immunized with BSA-STa_A14T_ or 3xSTa_N12S_-mnLT_R192G_/_L211A_ double mutant LT monomer (mnLT_R192G_/_L211A_ was created by fusion of a mutant LTA subunit to a single LTB subunit to obtain a single peptide) developed similar levels of anti-STa antibodies. Pigs were also immunized and the derived antibodies evaluated for efficacy to passively provide protection against ETEC diarrhea using a pig model. Piglets with passively acquired antibodies induced by the genetic fusion protein were better protected against a STa-positive ETEC strain challenge. Previously, Nandre et al. (2017) had demonstrated that toxoid fusion 3xSTa_N12S_-dmLT induced neutralizing antitoxin antibodies in intraperitoneally or subcutaneously immunized mice [67]. Pregnant gilts immunized intramuscularly with the toxoid and the suckling piglets were then challenged with a STa-positive ETEC strain. The protective efficacy of passively acquired antitoxin antibodies against ETEC diarrhea was assessed. All three immunized gilts developed anti-STa IgG and IgA antibodies and piglets born to the immunized dams acquired anti-STa and anti-LT antibodies. A challenge with a STa-positive ETEC strain did not provoke watery diarrhea in any piglets born to the immunized dams (20 remained normal and 8 piglets developed mild diarrhea compared to unimmunized control animals where 26/32 piglets developed watery diarrhea). Thus, both studies indicated that passively acquired anti-STa antibodies were protective against ETEC diarrhea.

In a study by Ruan et al. (2011), nucleotides encoding peptides for F4 (FaeG), F18 (FedF), and LT toxoid (LT_R192G_) were genetically fused to obtain a tripartite adhesion-adhesin-toxoid chimeric antigen [62]. The data showed that FaeG-FedF-LT_R192G_ A2:B fusion elicited anti-F4ac, anti-F18, and anti-LT antibodies in intaperitoneally immunized mice and pigs. Porcine antibodies neutralize CT and inhibited adherence of both F4 and F18 fimbriae, in vitro. Immunized piglets were protected against a challenge with a F4ac/LT/STb ETEC strain. The construction elicited antibodies causing a 2- to fivefold reduction in adherence by both F4ac and F18 fimbriae. Non-vaccinated piglets developed severe diarrhea and dehydration after the challenge. This study proved that multiple adhesion antigens and multiple toxins antigens could be expressed by a single protein. In the future, the expression of a tripartite antigen by a non-pathogenic *E. coli* field isolate could also lead to the development of a live attenuated vaccine strain that could be use against porcine ETEC.

The STb gene is highly prevalent in *E. coli* strains isolated from pigs with PWD and it is an important virulence factor [57]. The majority of ETEC strains causing porcine diarrhea, especially PWD, produce LT and STb. Data from recent studies indicate that LT_R192G_ toxoid and STb fusion antigen (LT_R192G_-STb) elicited protective anti-LT and anti-STb antibodies in pigs [28]. These researchers used LT_R192G_ derived from porcine ETEC to carry mature STb peptide (LT192-STb) to enhance STb immunogenicity. Anti-LT and anti-STb antibodies were produced in immunized rabbits and pigs. In a challenge with a STb-positive ETEC strain, all 10 suckling piglets born by immunized gilts remained healthy whereas 7/9 piglets born by non-immunized gilt developed moderate diarrhea. Rabbit anti-LT antibodies neutralized CT in vitro as the intracellular cAMP levels in T84 cells was not increased. Anti-STb antibodies tested in loop assay with a mixture of culture filtrate of a test strain F4ac/STb and serum or fecal sample from immunized rabbits had significantly less fluid accumulated compared to loops incubated with the culture filtrate of the same strain. The authors hypothesized that fusing STb at the C-terminus of LT_R192G_ with a longer hinge could display STb antigen better. In fact, in rabbits immunized with pLT_R192G_-L-linker-STb, fusion had a significantly greater level of anti-LT IgG and anti-STb IgG antibodies, but not anti-STb IgA antibodies. Also, anti-STb antibodies obtained following immunization with 6xHis-tagged pLT_R192G_-Gly-Pro-STb fusion antigens were protective against STb toxin. In this case, a STb toxoid was not obtained, the molecule being fully toxic. Nevertheless, in that specific study, it did not seem to affect the animal receiving the vaccine. Further work is required to find a reliable non-toxic STb fragment or mutant. We have to remember that truncating STb may reduce its toxicity but it may also render the molecule incapable of inducing neutralizing antibodies (Dubreuil et al., 1996).

A trivalent enterotoxin fusion protein (STa-LTB-STb) for vaccination purpose was constructed as a single toxoid [63]. The toxicity of STa was diminished by a mutation at one disulfide bridge but the toxicity of STb was intact. In mice, this fusion protein elicited significant antibody responses to LTB, STa, and STb able to neutralize the biological activity of both STa and STb. After intraperitoneal challenge with an ETEC strain, the mortality in immunize mice was significantly lower than the control cohorts (*p* < 0.01).

Evaluation of STa-LTB-STb with F4ac and F5 antigens as a novel multivalent vaccine candidate was carried out in a pig model [47]. IgG titers in serum as well as colostrum in all vaccinated sows were significantly higher than in the control group (*p* < 0.05). Piglets in the vaccinated group exhibited healthier status than the non-immunized group. In a F4-positive ETEC challenge, none of the vaccinated piglets experienced diarrhea.

As F4/LT/STb and F18/STa/STb/Stx2e are the predominant ETEC pathotypes causing PWD in weaned pigs [57], Lu T. et al. (2020) recently explored a novel epitope- and structure-based vaccinology platform called multiepitope-fusion-antigen (MEFA) for vaccine development [68]. As MEFA-based vaccine does not carry somatic antigens (somatic proteins, LPS), it is less likely to cause associated side effects. The MEFA technology had been applied first for human ETEC [69, 70]. Assisted by protein modeling and molecular dynamics simulation, MEFA identifies a backbone immunogen. By epitope substitution of LT toxoid as the backbone to present neutralizing epitopes of two ETEC fimbriae (F4 and F18) and four toxins (LT, STa, STb, and STx2e), a PWD fimbria-toxin MEFA was generated to mimic epitope native antigenicity. Mice subcutaneously immunized with PWD MEFA protein develop strong responses to F4, F18, LT, and STb and moderate response to STx2e and STa toxins. MEFA-induced antibodies inhibited adherence of F4 or F18 fimbrial bacteria to pig intestinal cells and also neutralized toxicity of all four enterotoxins. These results strongly suggest a potential application of this MEFA protein in developing a protective PWD vaccine with broad action. This study also demonstrated that neutralizing epitopes from ETEC virulence determinants in pig PWD can be integrated into a single immunogen. To the authors’ knowledge, this was the first report of an antigen or vaccine candidate inducing antibodies against all ETEC virulence factors associated with pig PWD. Future pig immunization and challenge studies are needed to verify MEFA-induced protective efficacy against PWD. The authors also suggested that a host strain or vector system be used to effectively express and secrete MEFA protein onto the membrane to optimize an oral vaccine format, as parenteral vaccines are not desirable for young animals due to concerns of cost-effectiveness and the need of adjuvants and booster administration.

## Conclusion

After many years of research acquiring knowledge on ETEC virulence factors and evaluating vaccine preparations, these bacterial pathogens remain a leading cause of diarrhea in pig herds [71]. In fact, although there is adhesion-based vaccines that provide some protection, there is no universal protective ETEC vaccine commercially available against ETEC diarrhea [14]. As discussed, some ETEC strains harbor one or more enterotoxins but lack any of the known fimbrial or non-fimbrial adhesins. For this reason, effective ETEC vaccines would benefit from the inclusion of enterotoxin antigens. Thus, the search for a new generation of cost-effective and broadly protective vaccine against porcine neonatal and specifically PWD is pursued.

As adhesins and enterotoxins are critical virulence determinants of ETEC in porcine diarrhea, vaccines inducing anti-adhesin combined with anti-toxin immunity are now foreseen as a promising approach to improve protection against ETEC diarrhea. Incorporation of heat-labile and heat-stable enterotoxins in a single molecule together with fimbrial epitopes designed to block attachment of ETEC to mucosal surfaces and neutralize the noxious activities of enterotoxins need to be considered. Nevertheless, other factors influence the success or failure of a vaccine, like immunization procedure and animal genetics [72] and these have also to be taken into account.

Oral immunization although efficient for neonatal diarrhea has limitations when PWD is considered as this strategy represents a logical route required to stimulate sIgA production. On the other hand, certain parenteral immunization strategies are encouraging although some dilemmas associated with this approach such as the cost and the stress response induced in piglets as a result of required repeated injection.

Overall, some studies have shown the potential of associating multiple antigens found in ETEC (Table 1). In order to confirm or infirm these results, future studies of vaccine preparations developed against ETEC-provoked diarrhea with larger sampling sizes will have to be conducted.

**Table 1:** Various vaccine preparations developed to control ETEC-provoked diarrhea, indicating the immunized animal model and the administration route

	Immunized animal	Vaccinal preparation	Reference
Oral route			
	Mice	LT_R192G_-STa_A13Q_	Liu et al., 2015
		LT_R192G_-STb	
	Piglets	FaeG-FedF-LT_R192G_A_2_:5LTB	Ruan and Zhang, 2013
	Piglets	F4ac-LT_R192G_-STb	Ruan et al., 2011
	Mice	F41-LT_R192G_-STa_A13Q_	Liu et al., 2015
		F41-LT_R192G_-STb	
	Mice	F4ac-STa-LTB-STb	You et al., 2011
	Mice	LTA-STa_A13Q_-STb-LTA2-LTB-STa_A13Q_-STb	Feng and Guan, 2019
Parenteral route			
	Rabbits (IM)	pLT_R192G_:STa_N11K_	Zhang et al., 2010
	Sows (IM)	pLT_R192G_:STa_P12F_	
		pLT_R192G_:STa_A13Q_	
	Mice (SC)	BSA-STa_A14T_	Seo et al., 2019
		3xSTa_N12S_-mnLT_R192G/L211A_	
	Mice (IP or SC)	3xSTa_N12S_-dmLT_R192G/L211A_	Nandre et al., 2017
	Pregnant gilts (IM)		
	Mice and pigs (IP)	FaeG-FedF-LT_R192G_-A2:B	Ruan et al., 2011
	Rabbits and pregnant gilts (IM)	pLT_R192G_-L-linker-STb	Zhang and Francis, 2010
		6xHis-tagged pLT_R192G_-Gly-Pro-STb	
	Mice (IP)	STa-LTB-STb	You et al., 2011
	Pig (IM)	F4ac-STa-LTB-STb	Zhang et al., 2018
		F5-STa-LTB-STb	
	Mice (SC)	F4-LT-STa-STb-STx2e	Lu et al., 2020
		F18-LT-STa-STb-STx2e	

*IM* intramuscular, *IP* intraperitoneal, *SC* subcutaneous
